# Nebulised heparin as a treatment for COVID-19: scientific rationale and a call for randomised evidence

**DOI:** 10.1186/s13054-020-03148-2

**Published:** 2020-07-22

**Authors:** Frank M. P. van Haren, Clive Page, John G. Laffey, Antonio Artigas, Marta Camprubi-Rimblas, Quentin Nunes, Roger Smith, Janis Shute, Mary Carroll, Julia Tree, Miles Carroll, Dave Singh, Tom Wilkinson, Barry Dixon

**Affiliations:** 1 Australian National University, Medical School, Canberra, Australia; 2 Intensive Care Unit, the Canberra Hospital, Canberra, Australia; 3 Sackler Institute of Pulmonary Pharmacology, King’s College London, London, UK; 4 Anaesthesia and Intensive Care Medicine, School of Medicine, and Regenerative Medicine Institute (REMEDI) at CÚRAM Centre for Research in Medical Devices, Biomedical Sciences Building, National University of Ireland Galway, Galway, Ireland; 5 Department of Anaesthesia, University Hospital Galway, Saolta Hospital Group, Galway, Ireland; 6 Critical Center, Corporació Sanitaria Parc Tauli , CIBER Enfermedades Respiratorias, Autonomous University of Barcelona, Sabadell, Spain; 7 Institut d’Investigació I Innovació Parc Tauli (I3PT), CIBER de Enfermedades Respiratorias, Sabadell, Spain; 8 Institute of Systems, Molecular and Integrative Biology, University of Liverpool, Liverpool, UK; 9 Department of Critical Care Medicine, St Vincent’s Hospital, Melbourne, Australia; 10 School of Pharmacy and Biomedical Science, University of Portsmouth, Portsmouth, UK; 11 Department of Respiratory Medicine, University of Southampton, Southampton, UK; 12 National Infection Service, Public Health England, Porton Down, UK; 13 Medicines Evaluation Unit, University of Manchester, Manchester, UK

**Keywords:** COVID-19, ARDS, SARS, Nebulised heparin, Unfractionated heparin, SARS-CoV-2

## Abstract

Nebulised unfractionated heparin (UFH) has a strong scientific and biological rationale and warrants urgent investigation of its therapeutic potential, for COVID-19-induced acute respiratory distress syndrome (ARDS). COVID-19 ARDS displays the typical features of diffuse alveolar damage with extensive pulmonary coagulation activation resulting in fibrin deposition in the microvasculature and formation of hyaline membranes in the air sacs. Patients infected with SARS-CoV-2 who manifest severe disease have high levels of inflammatory cytokines in plasma and bronchoalveolar lavage fluid and significant coagulopathy. There is a strong association between the extent of the coagulopathy and poor clinical outcomes.

The anti-coagulant actions of nebulised UFH limit fibrin deposition and microvascular thrombosis. Trials in patients with acute lung injury and related conditions found inhaled UFH reduced pulmonary dead space, coagulation activation, microvascular thrombosis and clinical deterioration, resulting in increased time free of ventilatory support. In addition, UFH has anti-inflammatory, mucolytic and anti-viral properties and, specifically, has been shown to inactivate the SARS-CoV-2 virus and prevent its entry into mammalian cells, thereby inhibiting pulmonary infection by SARS-CoV-2. Furthermore, clinical studies have shown that inhaled UFH safely improves outcomes in other inflammatory respiratory diseases and also acts as an effective mucolytic in sputum-producing respiratory patients. UFH is widely available and inexpensive, which may make this treatment also accessible for low- and middle-income countries.

These potentially important therapeutic properties of nebulised UFH underline the need for expedited large-scale clinical trials to test its potential to reduce mortality in COVID-19 patients.

## Introduction

In December 2019, a novel coronavirus (severe acute respiratory syndrome coronavirus 2, SARS-CoV-2) emerged in China and has since spread globally. A large proportion of patients admitted to hospital for coronavirus disease 2019 (COVID-19) develop acute respiratory distress syndrome (ARDS) criteria according to the Berlin definition [1–3]. ARDS is an acute inflammatory lung injury, associated with increased pulmonary vascular permeability, increased lung weight and loss of aerated lung tissue, affecting 23% of mechanically ventilated critically ill patients. The hospital mortality of ARDS is estimated between 35 and 46% depending on ARDS severity [4, 5]. However, the death rate in COVID-19 patients with ARDS appears to be higher, up to 66% [2]. It has been suggested that COVID-19 pneumonia-associated ARDS is a specific disease or perhaps a specific phenotype of ARDS, whose distinctive features are severe hypoxaemia initially associated with relatively well-preserved lung mechanics [6, 7]. A possible explanation for such severe hypoxaemia occurring in compliant lungs is the loss of lung perfusion regulation and hypoxic vasoconstriction. In addition, COVID-19 ARDS patients have higher plasma markers of coagulation, such as D-dimers, increased prothrombin time and a lower platelet count [2, 8–12]. Endothelial dysfunction and microvascular thrombosis could therefore also explain the specific pulmonary findings in severe COVID-19—high dead space and impaired oxygenation in the absence of significant decrease in pulmonary compliance. Post-mortem studies and lung biopsies of SARS-CoV-2 patients with ARDS indeed demonstrated pulmonary fibrin deposition with hyaline membranes in the alveolar spaces and extensive pulmonary microvascular thrombi [13–15].

Pulmonary disease severity is also related to an aggressive host inflammatory response to SARS-CoV-2 infection, with release of an uncontrolled cytokine storm inflicting damage to other organs including the cardiac, hepatic and renal systems [16].

In this focused review, we present the biological and scientific rationale for the use of nebulised UFH for COVID-19 pneumonia and ARDS in hospitalised patients and make a call for an urgent, global approach to the investigation of its therapeutic potential for this devastating condition.

## Biological rationale: pathophysiology of COVID-19

**Fig. 1:**
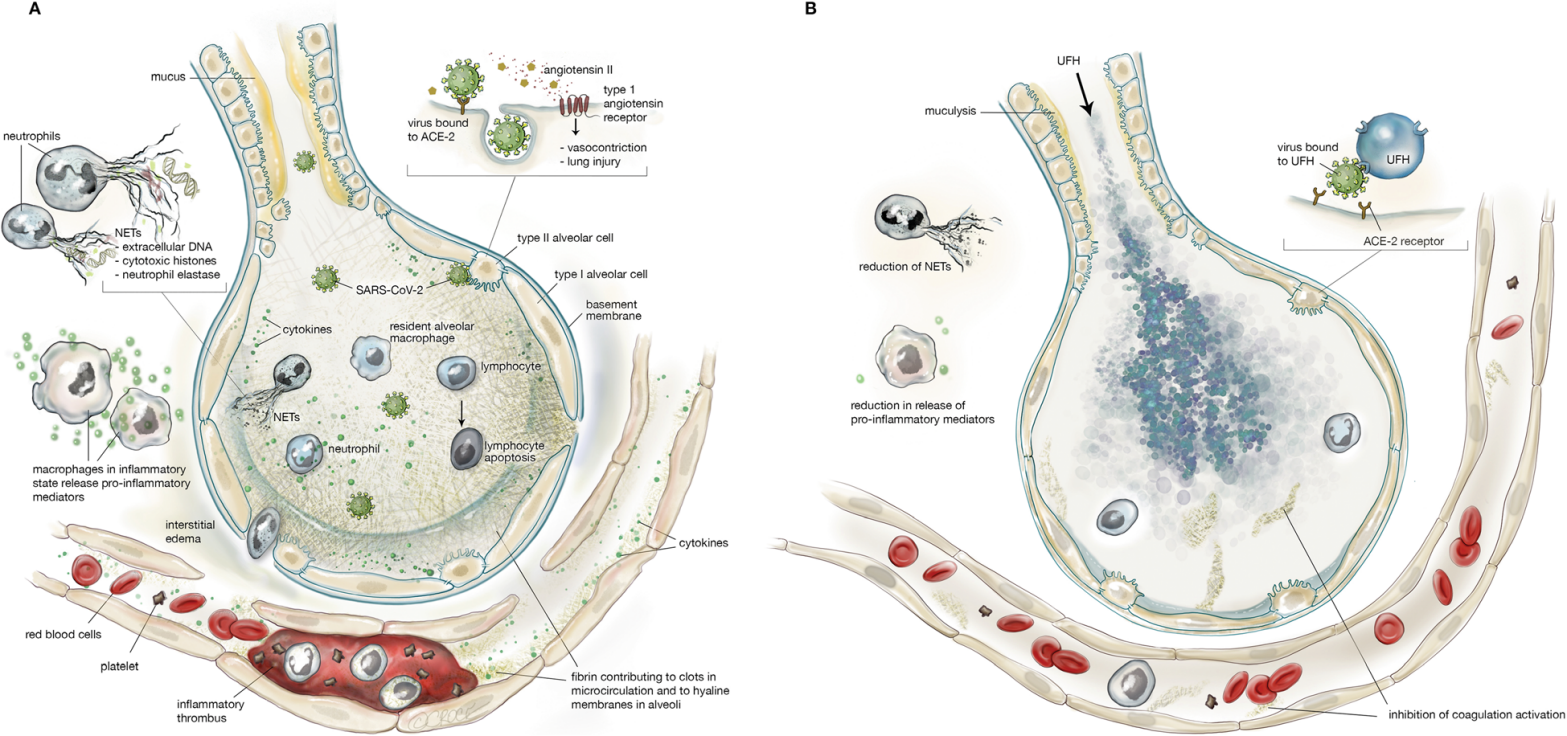
**a** Lung injury in coronavirus disease 2019 (COVID-19). Severe acute respiratory syndrome coronavirus 2 (SARS-CoV-2) binds to angiotensin-converting enzyme 2 (ACE-2) primarily on type II alveolar cells. After endocytosis of the viral complex, surface ACE-2 is downregulated, resulting in unopposed angiotensin II accumulation. SARS-CoV-2 further causes lung injury through activation of residential macrophages, lymphocyte apoptosis and neutrophils. The macrophages produce cytokines and chemokines, resulting in a cytokine storm. Inflammatory exudate rich in plasma-borne coagulation factors enters the alveolar space, followed by expression of tissue factor by alveolar epithelial cells and macrophages and the formation of fibrin and the hyaline membrane. Neutrophils in the alveoli cause formation of NETs, composed of extracellular DNA, cytotoxic histones and neutrophil elastase, which cause further lung injury. COVID-19 also induces microvascular endothelial damage leading to increased permeability, expression of tissue factor with coagulation activation and thrombus formation. **b** Proposed effects of inhaled nebulised unfractionated heparin (UFH) in COVID-19 lung injury. UFH prevents SARS-CoV-2 from binding to ACE-2 and from entering the alveolar cells. UFH reduces formation of the hyaline membrane and microvascular thrombosis, counteracts the hyperinflammation and the formation of NETs, increases NO release with vasodilation and also has mucolytic properties. NETs, neutrophil extracellular traps; SARS-CoV-2, severe acute respiratory syndrome coronavirus 2; ACE-2, angiotensin-converting enzyme 2; COVID-19, coronavirus disease 2019. Permission was granted by © Beth Croce, Bioperspective.com to reuse this figure

The pathophysiology of COVID-19 associated ARDS is summarised in Fig. 1a and is characterised by diffuse alveolar damage, hyperinflammation, coagulopathy, DNA neutrophil extracellular traps (NETS), hyaline membranes and microvascular thrombosis.

### Infection, inflammation and coagulopathy

SARS-CoV-2 binds to angiotensin-converting enzyme-2 (ACE-2) to gain cellular entry. ACE-2 is widely expressed in the lungs, predominantly on alveolar type II epithelial cells, but also on bronchial epithelial cells and on arterial and venous endothelial cells [17, 18]. ACE-2 hijacking prevents angiotensin II degradation. Angiotensin II signals through the type 1 angiotensin receptor, causing vasoconstriction and lung injury, including endothelial injury leading to tissue factor expression and coagulation cascade activation [19]. Widespread endothelial inflammation and apoptosis leading to endothelial dysfunction in multiple organs are associated with direct viral infection of endothelial cells in COVID-19, as well as immune-mediated responses to infection of pulmonary alveolar cells, and a pro-coagulant state [20].

Patients with SARS-CoV-2 who manifest severe disease, including ARDS, multi-organ failure, and death, have higher plasma and BALF levels of inflammatory cytokines (‘cytokine storm’); higher plasma markers of coagulation, such as D-dimers; and increased prothrombin time and a lower platelet count [2, 8–11, 21–23]. An aggressive dysfunctional inflammatory response following pyroptosis of virus-infected cells is strongly implicated in damage to the lungs [16]. For example, plasma concentrations of a range of pro-inflammatory cytokines were higher in both ICU patients and non-ICU patients infected with SARS-CoV-2 than in healthy adults, and some of these agents were also higher in ICU patients than non-ICU patients [9]. Furthermore, elevated plasma IL-6 was reported to be a predictor of fatality, suggesting that mortality might be driven by virally induced hyperinflammation [21, 22]. The expression of a large number of cytokines is also elevated in BALF samples from COVID-19 patients compared to control [23]. Finally, intravascular DNA neutrophil extracellular traps (NETs) have been reported in COVID-19 patients, where they may contribute to cytokine release, coagulopathy and respiratory failure [24, 25].

### Hyaline membrane formation

COVID-19 is associated with the development of ARDS displaying the typical features of diffuse alveolar damage [26–30]. The hallmark histological feature of ARDS is a fibrin mesh in the air sacs, known as a hyaline membrane, on which leucocytes attach and manifest the inflammatory responses that result in diffuse alveolar damage. Hyaline membrane formation is a consistent and early manifestation of the inflammatory response in ARDS [27, 30–33]. Hyaline membrane formation results from entry into the alveolar space of inflammatory exudate that is rich in plasma-borne coagulation factors. The subsequent expression of tissue factor by alveolar epithelial cells and macrophages triggers the conversion of these coagulation factors to fibrin and the formation of the hyaline membrane [34]. In pro-inflammatory conditions, alveolar epithelial cells and macrophages also express plasminogen activator inhibitor-1, which prevents the removal of this membrane through endogenous fibrinolysis [32, 35]. Pulmonary coagulation is evident in increased markers of thrombin generation, soluble tissue factor and factor VIIa activity found in bronchoalveolar lavage fluid (BALF) from ARDS patients, together with an increased release of plasminogen activator inhibitor-1 [36].

Hyaline membrane formation may contribute to lung injury through a number of mechanisms. The hyaline membrane forms a physical barrier thereby limiting the diffusion of gases. Alveolar compliance and the action of surfactant are also limited by fibrin formation in the alveoli contributing to atelectasis, and finally, the laying down of a fibrin matrix may promote subsequent lung fibrosis [32, 37].

### Microvascular thrombosis

Fibrin accumulation in pulmonary capillaries and venules, which leads to microvascular thrombosis, is an early feature of ARDS and the extent of this fibrin accumulation correlates with the severity of lung injury [38–41]. In response to inflammatory cytokines, the pulmonary capillary beds, venules and arterioles express tissue factor on endothelial cells and this triggers the conversion of plasma coagulation factors to fibrin [42]. Cytokine activation of NETosis and the presence of intravascular NETs are further associated with the initiation of thrombosis in arteries and veins, and NETs circulating at high levels in COVID-19 can trigger micro-embolic occlusion of small blood vessels in the lungs, heart and kidneys [24, 25].

Extensive microvascular thrombosis has been demonstrated in histological studies of ARDS [39, 40]. Angiographic studies showed the extent of microvascular obstruction correlated with the severity of respiratory failure and with mortality [38, 39]. Microvascular thrombosis increases lung dead space and the increase in dead space or its bedside surrogate ventilatory ratio was shown to be an independent marker of mortality in ARDS [43, 44]. Microvascular thrombosis also causes increased pulmonary vascular resistance, which may result in right heart failure [45].

There is a strong association between the extent of the coagulopathy and poor clinical outcomes. In a case series of 183 COVID-19 patients, those who died had markedly elevated D-dimers, elevated fibrin degradation products, longer prothrombin time and activated partial thromboplastin time compared to survivors on admission, often meeting criteria for disseminated intravascular coagulation [46]. Similar coagulation abnormalities were described in other case series and elevated D-dimer levels were associated with clinical outcomes [2, 47, 48]. In a Dutch case series of 184 COVID-19 positive patients, all of which received pharmacological thromboprophylaxis, the cumulative incidence of a composite outcome comprised of symptomatic pulmonary embolism (PE), deep-vein thrombosis, ischemic stroke, myocardial infarction, or systemic arterial embolism was 49%. The majority of thromboembolic events were PE (87%) [49]. Another recent case series showed that COVID-19 ARDS patients developed significantly more thrombotic complications than non-COVID-19 ARDS patients, mainly PE [12].

### Mucus exudates and DNA NETs

Excessive sputum production is a feature of approximately 30% of COVID-19 patients and the bronchi become filled with desquamated epithelial cells, mucus and thick mucus plugs [9, 50, 51]. Diapedesis of neutrophils into the alveolar space is proposed to be a source of excess NETs, composed of extracellular DNA and bound basic proteins including cytotoxic histones and neutrophil elastase, which are involved in both the generation of NETs and damage to pulmonary tissue [25]. Such NETs may further impair gas exchange and facilitate secondary infections. Intra-alveolar NETS are a feature of pneumonia-associated ARDS [52] and are likely to be present in the airways of COVID-19 patients with ARDS.

## Biological rationale: effects of nebulised UFH

The effects of nebulised UFH in COVID-19 are summarised in Fig. 1b. Nebulised UFH has anti-viral, anti-coagulant, anti-inflammatory and mucolytic effects.

### Anti-viral effects

Heparin is a member of a family of glycosaminoglycan molecules that include heparan sulphate, chondroitin sulphate, keratan sulphate and hyaluronic acid. These molecules are expressed throughout the body, with diverse biological roles, and are usually associated with respiratory and endothelial cell surfaces, basement membrane and extracellular matrices [53]. In humans, heparin is produced solely by mast cells and is stored in granules, where it makes up 30% of the dry weight of mast cell granules [54]. There is evidence that heparin plays a role in host defence. Firstly, mast cells are mostly located along blood vessels and are particularly associated with capillaries and post-capillary venules [55]. Secondly, organs exposed to the external environment, such as the lungs and gut contain a large proportion of the body’s mast cells [56]. Thirdly, heparin is conserved across a variety of different species, some of which do not have a blood coagulation system like ours (e.g. molluscs), suggesting heparin has significant biological roles unrelated to coagulation [57].

A large number of bacterial and viral pathogens depend upon interactions with proteoglycan molecules such as heparan sulphate, which is expressed on a range of human tissue surfaces, for adhesion and invasion of host tissues [53]. Several studies found heparin competes with heparan sulphate for bacterial and viral adhesion and may therefore limit pathogen invasion [58, 59]. For example, heparin limits adhesion of *Pseudomonas aeruginosa*, *Burkholderia cenocepacia*, *Burkholderia pseudomallei*, *Legionella pneumophila*, *Staphylococcus aureus*, *Streptococcus pyogenes*, *Streptococcus pneumoniae*, respiratory syncytial virus and influenza A [60–64]. Human and animal studies suggest these actions may reduce the development of pneumonia and bacteraemia [58, 65].

Previous studies demonstrated that UFH prevented SARS-associated coronavirus and other enveloped viruses such as human immunodeficiency virus and herpes simplex virus, from attaching to and invading mammalian cells [66–72]. A recent study demonstrated that the SARS-CoV-2 Spike S1 protein receptor-binding domain attaches to UFH and undergoes conformational change that may prevent it from binding ACE-2 as a result [73]. Importantly, the binding of heparin to the receptor-binding domain of the SARS-CoV-2 Spike S1 protein is orders of magnitude stronger for full-chain length heparin than low-+molecular weight heparins (LMWHs) [74]. This anti-viral effect of heparin has recently been confirmed in initial studies performed by Public Health England where an UFH preparation produced a concentration-dependent inhibition of SARS-CoV-2 infection of Vero E6 cells that was more active than LMWH, further suggesting that UFH may prevent invasion of pulmonary epithelium and vascular endothelium (M Carroll and J Tree, personal communication from Public Health England). The high concentration of SARS-Cov2 in the upper airways of COVID-19 patients and the above anti-viral properties of heparin makes the nebulised route of administration a unique and possibly effective treatment for COVID-19.

### Anti-inflammatory effects

Heparin also has other pharmacological actions of potential benefit including inhibition of inflammatory cytokines implicated in COVID-19 and the inhibition of inflammatory cell recruitment into tissues via blocking many of the key adhesion molecules expressed on vascular endothelium, improvement in lung function and increased nitric oxide release [60, 75–78]. Heparin has been shown to reduce the expression of pro-inflammatory mediators in human alveolar macrophages injured by lipopolysaccharide and to decrease the NF-kB pathway in alveolar cells [79]. Furthermore, nebulised heparin decreases pro-inflammatory cytokines in lung tissue and the expression of NF-kB and TGF-β effectors in alveolar macrophages [79, 80]. Heparin, through multiple actions including inhibition of adhesion molecules and heparanase activity, has also been shown to reduce the infiltration of inflammatory cells into a range of tissues, including the lung, activities that are independent of its anti-coagulant properties [78]. Additionally, heparin is known to have important inhibitory effects on the complement cascade that has also been implicated in the vascular injury associated with COVID-19 [78]. In pre-clinical animal models, UFH was a more effective anti-inflammatory agent than LMWHs, which may be an important additional pharmacological property of this drug in the context of the hyperinflammatory state associated with COVID-19 [78, 81].

Overall, the multiple pharmacological properties of UFH may be important in the context of treating the hyperinflammatory state associated with COVID-19, particularly in the absence of clear evidence of the efficacy of other anti-inflammatory therapies [82].

### Anti-coagulant effects

Heparin’s anti-coagulant properties have been used in clinical practice to limit systemic fibrin deposition since 1935 [83]. Heparin inhibits coagulation activation through a range of mechanisms, including catalysing the action of antithrombin, promoting tissue factor pathway inhibitor expression, reducing tissue factor expression and increasing endothelial expression of heparan sulphate, and through release of tissue plasminogen activator by the endothelium.

Nebulised UFH targets pulmonary fibrin deposition and inflammation, and local administration to the lungs allows higher dosages and increases local efficacy, reduces the risk of systemic bleeding and is more effective than intravenous administration [84, 85]. Importantly, previous studies have shown that following nebulisation, UFH does not enter the systemic circulation significantly which means it can be used in addition to systemic therapeutic or prophylactic anti-coagulation without concerns of furthering systemic anti-coagulation. The use of nebulised UFH in other respiratory settings was not associated with local side effects in the lung including bleeding [85–89].

### Mucolytic effects

Mucus obstruction of the airways is compounded by the presence of DNA NETs in inflammatory lung diseases such as cystic fibrosis (CF), asthma, COPD and ARDS [90]. DNA contributes to sputum elasticity and reduced cough clearance, and in CF sputum, heparin disaggregated DNA/actin bundles and activated endogenous DNase to reduce sputum elasticity [91]. When DNA NETS are broken down, the potential for the release of cytotoxic histones, neutrophil elastase and IL-8 encrypted by the DNA is mitigated by the ability of heparin to neutralise these basic proteins [90]. Independently of the presence of DNA NETs, electrostatic mucin interactions and viscosity are increased by a low pH in airway surface liquid, as seen in cystic fibrosis (CF), asthma, COPD and ARDS and these effects are also reversed by heparin [92, 93]. The mucolytic properties of heparin have been utilised in the treatment of CF patients with no safety issues and in particular inhaled nebulised UFH has been used safely in patients who are also receiving system anti-coagulation [94].

## Pre-clinical and clinical evidence in lung injury

Animal studies of nebulised UFH in different acute lung injury models have consistently shown a positive effect on pulmonary coagulation, inflammation and oxygenation (Table 1). Small human studies indicate that nebulised heparin limits pulmonary fibrin deposition, attenuates progression of acute lung injury and hastens recovery (Table 2) [95, 96]. In smoke inhalation-related lung injury, pre-clinical and clinical studies have suggested that administration of inhaled anti-coagulants improves oxygenation, reduces lung injury severity and improves survival without altering systemic markers of clotting and anti-coagulation [97].

**Table 1:** Pre-clinical studies of nebulised heparin treatment for acute lung injury

Dosage (Timing)	Species	Model (Sacrifice)	Nebulizer	Outcomes	Side effects	Reference
**Animal models of acute lung injury treated with nebulised heparin**						
1000 IU/kg (30min before injury and every 6h)	Rat	it. Streptococcus pneumoniae (40h)	Aeroneb Pro Nebulizer	↓ Pulmonary coagulation	NR	Hofstra et al, 2009 [104]
1000 IU/kg (30min before, 6h and 12h after injury)	Rat	iv. LPS (7.5 mg/kg) (16h)	Aeroneb Pro Nebulizer	↓ Coagulation	NR	Hofstra et al, 2010 [105]
1000 IU/kg (30min before injury and every 6h)	Rat	it. Pseudomonas aeruginosa (16h)	Aeroneb Pro Nebulizer	=	NR	Cornet et al, 2011 [106]
Dose NR (5 min after injury)	Mouse	inh. Chlorine (400 ppm for 30min) (6h)	AirLife Brand Misty Max 10	↓ Decreased inflammation	No side effects	Zarogiannis et al, 2014 [107]
1000 IU/kg (4h and 8h after injury or 30min before, 4h and 8h after injury)	Rat	it. LPS (10μg/g) (24h)	Aeroneb Pro Nebulizer	↓ Pulmonary coagulation and inflammation	NR	Chimenti et al, 2017 [80]
**Animal models of acute lung injury treated with nebulised heparin and combined with another treatment**						
10000 IU (1h after injury, every 4h)	Sheep	Smoke inh. and it. Pseudomonas aeruginosa (24h)	AirLife Brand Misty Max 10	↓ Lung injury and airways obstruction ↑ PaO2	No side effects	Murakami et al, 2002 [108]
10000 IU (30min after injury, every 4h for 24h) or combined with intravenous 10 mg/kg/h lisofylline	Sheep	Smoke inh. (48h)	AirLife Brand Misty Max 10	↓ Need for MV ↑ PaO2	No side effects	Tasaki et al, 2002 [109]
10000 IU (2h after injury, every 4h) or combined with nebulized 290 IU recombinant antithrombin	Sheep	Cutaneous burn and smoke inh. (48h)	AirLife Brand Misty Max 10	Combination: ↓ Pulmonary inflammation and airways obstruction ↑ PaO2	No side effects	Enkhbataar et al, 2007 [110]
10000 IU (1h after injury, every 4h) combined with intravenous 0.34 mg/kg/h recombinant antithrombin	Sheep	Cutaneous burn and smoke inh. (48h)	AirLife Brand Misty Max 10	↓ Inflammation, oedema, airways obstruction ↑ PaO2	No side effects	Enkhbataar et al, 2008 [111]
10000 IU (2h after injury, every 4h) combined with intravenous 6 IU/kg/h recombinant antithrombin (from 1h after injury until the end of the study) and nebulized 2 mg tissue plasminogen inhibitor (4h after injury, every 4h)	Sheep	Cutaneous burn and smoke inh. (48h)	AirLife Brand Misty Max 10	↓ Lung injury, oedema and airways obstruction ↑ PaO2	No side effects	Rehberg et al, 2014 [112]
1000 IU/kg (4h, 12h and 28h after injury) combined with nebulized 500 IU/kg antithrombin (4h and 28h after injury)	Rat	it. HCl and LPS (30 μg/g) (72h)	Aeroneb Pro Nebulizer	↓ Pulmonary coagulation and inflammation	No side effects	Camprubí-Rimblas et al, 2020 [113]

↓: reduced, ↑: increased, =: equal, *inh* inhalation, *it.* intratracheal, *iv.* intravenous, *LPS* lipopolysaccharide, *MV* mechanical ventilation, *NR* not reported

**Table 2:** Clinical studies of nebulised heparin treatment for acute lung injury

Dosage (Timing)	Patients	n	Nebulizer	Outcomes	Reference
**Clinical studies of acute respiratory distress syndrome with nebulised heparin**					
50000-400000 IU/day (two days)	MV ARDS Open-label phase 1 trial	16	Aeroneb Pro Nebulizer	↓systemic coagulation (↓ pulmonary coagulation, 400000 IU)	Dixon et al, 2008 [114]
25000 IU (every 4h or 6h, max 14 days)	> 48h MV RCT	50	Aeroneb Pro Nebulizer	↓systemic coagulation ↑ Free days MV	Dixon et al, 2010 [100]
5000 IU (four times a day until cease MV or discharge from the UCI)	> 48h MV Phase 2 RCT	214	Aeroneb Pro Nebulizer	=	Bandeshe et al, 2016 [115]
50000 IU (one day)	Elective cardiac surgery RCT	40	Aeroneb Pro Nebulizer	↓ alveolar dead space fraction and tidal volumes	Dixon et al, 2016 [101]
25000 IU (every 6h, 10 days)	MV ARDS RCT	256	Aeroneb Pro Nebulizer	↓Lung injury ↑ 60 day survivors at home	Dixon et al, 2020 (submitted for publication)
**Clinical studies of acute respiratory distress syndrome with nebulised heparin and combined with another treatment**					
5000 IU combined with N-acetylcysteine and bronchodilator (every 4h for 7 days)	Burn patients paediatric Retrospective	90 (children)	NR	↓atelectasis, reintubation and mortality	Desai et al, 1999 [116]
5000 IU combined N-acetylcystine and bronchodilator (every 4h for 7 days)	Burn patients Retrospective	150 (children/adults)	NR	=	Holt et al, 2008 [117]
10000 IU combined N-acetylcystine and bronchodilator (every 4h for 7 days)	Burn patients Retrospective	30	NR	↓ lung injury ↓ mortality ↑ oxygenation	Miller et al, 2009 [118]
5000 IU combined N-acetylcystine and bronchodilator (every 4h for 7 days)	Burn patients Retrospective	63	NR	=	Yip et al, 2011 [119]
5000 IU combined N-acetylcystine and bronchodilator (every 4h for 7 days)	Burn patients Retrospective	40	NR	=	Kashefi et al, 2014 [120]
10000 IU combined N-acetylcystine and bronchodilator (every 4h for 7 days)	Burn patients Retrospective	72	NR	↑ free days MV	McIntire et al, 2017 [121]
25000 IU (every 4h for 14 days)	Burn patients RCT (terminated, insufficient recruitment of patients and high costs associated with the purchase and blinding of study medication)	160	Aeroneb Pro Nebulizer	Not available	Glas et al, 2014 [122]
**Clinical studies of COVID-19 with nebulised heparin**					
25000 IU (every 6h for 10 days)	SARS CoV 2 infection, on MV	RCT ACTRN: 12620000517976	Aeroneb Pro Nebulizer	On-going	Dixon et al, 2020 [123]
25000 IU (every 6h up to 21 days)	SARS CoV 2 infection, pre-ICU	RCT EudraCT: 2020-001736-95	Aeroneb Pro Nebulizer	On-going	ACCORD-2 (NHS UK)

↓: reduced, ↑: increased, =: equal, *MV* mechanical ventilation, *RCT* randomized controlled trial, *NR* not reported

Early-phase trials in patients with acute lung injury and related conditions found that nebulised heparin reduced pulmonary dead space, coagulation activation, microvascular thrombosis and deterioration in the Murray Lung Injury Score and increased time free of ventilatory support (Table 2) [98–102]. A multi-centre randomised double-blind placebo-controlled trial of nebulised heparin in 256 patients with or at risk of developing ARDS, investigated whether UFH accelerated recovery and has been completed (B Dixon personal communication, submitted for publication).

## Clinical evidence in SARS-CoV-2

Published data suggest that patients with SARS-CoV-2 treated with systemic UFH or LMWH had better clinical outcomes. For example, a non-randomised study found patients with sepsis-induced coagulopathy and D-dimer levels that were greater than 6-fold the upper limit of normal, were more likely to survive if administered heparin or LMWH [11]. In another observational study in 2773 patients hospitalised with COVID-19, mechanically ventilated patients who received systemic anti-coagulation during their hospital course had a lower hospital mortality (adjusted HR of 0.86 per day, 95% confidence interval 0.82–0.89, *p* < 0.001) [103]. This difference was not seen in all COVID-19 patients, suggesting that the beneficial effects may be more pronounced in patients with severe disease.

There are currently no published studies of nebulised heparin in COVID-19 patients, but there are several in preparation or being conducted (Table 2). In the UK, a study of nebulised UFH has been started under the national ACCORD programme (ACCORD 2: A Multicentre, Seamless, Phase 2 Adaptive Randomisation Platform Study to Assess the Efficacy and Safety of Multiple Candidate Agents for the Treatment of COVID 19 in Hospitalised Patients, EudraCT number 2020-001736-95). This study is investigating the effects of nebulised UFH administered 4 times daily in hospitalised patients testing positive for SARS-CoV-2, but before patients require ICU admission, on top of standard of care (Singh et al., personal communication). A multinational multi-centre randomised open-label clinical trial to determine if treatment with standard care and nebulised UFH, compared to standard care alone, reduces the duration of invasive mechanical ventilation in ICU patients with SARS-CoV-2 study is currently in preparation (Dixon and van Haren personal communication, ACTRN12620000517976).

There is an urgent need for more large-scale clinical trials to test whether nebulised UFH improves mortality in COVID-19 patients. Ideally, these studies should be linked together by a global network with the objective of standardising key outcomes, so a prospective individual patient meta-analysis (so called ‘meta-trial’) can be performed, to provide a rapid more generalisable answer to the question.

## Conclusion

Severe COVID-19 is characterised by diffuse alveolar damage, hyperinflammation, coagulopathy, DNA neutrophil extracellular traps (NETS) and microvascular thrombosis. There is a strong scientific and biological basis to test the use of nebulised UFH as a therapy for COVID-19 pneumonia and ARDS. UFH prevents SARS-CoV-2 from binding to ACE-2 and infecting cells and has relevant anti-coagulant, anti-inflammatory and mucolytic effects. Because of these multiple modes of action, inhaled UFH may offer clinical benefit across the time course of the disease. As an anti-viral, delivered via inhalation to the upper airways, the major point of entry of the virus, UHF may prevent infection and be a prophylactic treatment. If administered via nebulisation at the development of symptoms, its multiple properties may attenuate disease progression. As the disease progresses, UFH’s anti-inflammatory and anti-coagulant properties may be used to treat COVID-19-associated ARDS. In the pneumonic phase of COVID-19, which is typified by excess production of mucus, nebulised UFH’s known mucolytic effect can be used to aid recovery.

UFH is an inexpensive drug and widely available and its use as a potentially effective treatment for COVID-19 may have important humanitarian and economic implications especially for low- and middle-income countries.

The potential therapeutic properties underline the need for expedited large-scale clinical trials of nebulised UFH to test its potential to reduce mortality in COVID-19 patients.

## Data Availability

The datasets used for the current manuscript are available from the corresponding author on reasonable request.
